# Dissociating neural representations of multiple learned sensorimotor mappings

**DOI:** 10.3389/fnbeh.2026.1965610

**Published:** 2026-10-15

**Authors:** Antoine Caraballo, Sungshin Kim

**Affiliations:** 1 Department of Data Science, Hanyang University, Seoul, Republic of Korea; 2 Department of Artificial Intelligence, Hanyang University, Seoul, Republic of Korea; 3 Department of Healthcare Digital Engineering, Hanyang University, Seoul, Republic of Korea; 4 Center for Neuroscience Imaging Research, Institute for Basic Science, Suwon, Republic of Korea

**Keywords:** fMRI, multivariate pattern analysis, posterior parietal cortex, sensorimotor mappings, visuomotor adaptation, visuomotor components

## Abstract

**Introduction:**

Previous behavioral studies demonstrate that humans can simultaneously learn to adapt to multiple conflicting rules for how our physical movements are related to the sensory feedback we receive from our environment (sensorimotor mappings). The preparation of movement (motor planning), rather than the physical movement itself (motor execution), has been shown to play a crucial role in keeping these conflicting rules distinct in our memory. However, it remains unclear how the human brain represents distinct planning and execution processes for multiple sensorimotor mappings.

**Methods:**

To address this, we designed a functional magnetic resonance imaging (fMRI) experiment to dissociate planning from execution during the learning of multiple visuomotor mappings. Crucially, our design compared brain activity when participants planned for different visual outcomes. Specifically, participants simultaneously adapted to opposite rotational perturbations (+30° and −30°) during a visuomotor task but ultimately executed the exact same physical movement.

**Results:**

Our univariate results demonstrate that the BOLD activity increases in the posterior parietal cortex (PPC) during rotational planning, supporting its involvement in processing learned sensorimotor mappings. However, using multivoxel pattern analysis (MVPA) to decode neural patterns, we found a dissociation between motor execution and rotational planning. Although the PPC and cerebellum showed distinct decodable neural patterns for opposing motor execution, we did not observe significant decoding for opposing rotational planning.

**Discussion:**

This suggests that the PPC may not represent opposing motor plans as distinct, though we cannot rule out a lack of robust decoding power due to our low trial count.

## Introduction

Humans can simultaneously perform multiple motor tasks by learning distinct relationships between actions and their outcomes while building separate internal models (Wolpert and Kawato, 1998; Imamizu et al., 2003; Imamizu et al., 2004). Many real-world motor tasks are conflicting in that they often require identical actions for distinct outcomes or distinct actions for identical outcomes (Hirashima and Nozaki, 2012; Krasovsky et al., 2014). For example, consider riding a normal bicycle versus a “reversed” bicycle. Turning the handlebars to the left (identical action) turns the wheel of a normal bicycle to the left, but it turns the wheel of a reversed bicycle to the right (distinct outcomes). To successfully turn both bicycles to the left (identical outcome), we must turn the handlebars to the left on the normal bicycle, but to the right on the reversed bicycle (distinct actions).

Previous studies have demonstrated that conflicting sensorimotor mappings can be acquired distinctly with contextual cues despite initial interference through extensive training (Osu et al., 2004; Krakauer et al., 2006; Lee and Schweighofer, 2009; Woolley et al., 2007). Although conflicting sensorimotor mappings with similar task structures activate overlapping brain regions—such as when adapting to opposing rotational perturbations where visual feedback is shifted by an arbitrary degree—they can be successfully discriminated using patterns of fMRI activity in the sensorimotor system, including the cerebellum (Ogawa and Imamizu, 2013; Kim et al., 2015). In our previous experiment, these conflicting sensorimotor mappings were associated with both motor planning and its corresponding movement execution, without dissociating them (Kim et al., 2015). Dissociating these components is critical, as motor planning and execution appear to play distinct roles in motor adaptation. Furthermore, several behavioral studies have demonstrated a more significant role of planning over execution in the formation and retrieval of distinct motor memories (Hirashima and Nozaki, 2012; Sheahan et al., 2016; Lee et al., 2018).

We therefore aimed to further understand how motor planning and execution are distinctly represented in the brain during the acquisition of multiple sensorimotor mappings. Toward that goal, we deliberately designed a visuomotor task to isolate these neural processes. Specifically, participants concurrently adapted to three sensorimotor mappings indicated by distinct contextual color cues: one baseline mapping (baseline with 0° rotational perturbation) and two conflicting mappings (opposite rotational perturbations: +30° and −30°). To dissociate the neural representations of rotational planning (motor plan required to counteract the rotational perturbations) and motor execution, we combined these rotational perturbations with varying target positions (middle, high, low) across runs. For instance, to isolate rotational planning, target locations and rotational perturbations were offset to require different planned rotations for identical physical reach movements. In the same manner, holding target positions constant while varying the applied rotations isolated the execution of the movement. By analyzing the overlapping neural activity across specific pairs of runs sharing specific combinations of these factors, we attempted to successfully dissociate three fundamental components of visuomotor adaptation: (1) the motor plan generated to counteract the perturbation (referred to as rotational planning), (2) the physical execution of the movement (motor execution), and (3) the processing of the visual target location.

To isolate these three components, we first used general linear model analysis (GLM), followed by a conjunction analysis (intersection between activation maps across pairs of runs). Our univariate analysis identified brain regions with shared activation across different experimental conditions. Furthermore, we applied multi-voxel pattern analysis (MVPA) with a previously suggested cross-classification. By training the classifier on one set of conditions and testing on a different set of trials, we tested whether the neural patterns could generalize to a new context. The conjunction analysis and cross-classification ensured the dissociation of the invariant representation of planning, execution, and visual target location (Ogawa and Imamizu, 2013).

## Materials and methods

### Participants

We collected behavioral and fMRI data from 44 neurologically healthy participants (22 females; mean age = 24.6 years; range = 19–34 years) who provided written informed consent, with approval from the Sungkyunkwan University Institutional Review Board, and were compensated monetarily. All participants were assessed as right-handed by a modified version of the Edinburgh Handedness Inventory (Oldfield, 1971). All individuals had normal or corrected-to-normal vision and were unaware of the study’s purpose. We excluded 11 participants: one due to dizziness, eight due to excessive in-scanner movement, and two due to technical problems. In total, 33 participants were included in the data analysis (18 females; mean age = 24.5 years, range = 19–33 years).

### Apparatus and experimental setup

An MRI-compatible digitizing tablet was mounted on a dedicated pedestal resting comfortably over the participant’s lower abdomen. No direct support was provided for the participant’s reaching arm. Direct vision of the hand, pen, and tablet was completely obscured by the scanner bore and head coil. Participants relied exclusively on the visual feedback of the cursor projected on the screen.

### Visuomotor task

The experiment consisted of five fMRI runs, separated by short inter-run resting periods. The duration of the runs was approximately 14 min for Runs 1 and 2, 10 min for Run 3, 13 min for Run 4, and 10 min for Run 5. Prior to functional scanning, the participants completed 80 practice trials of the visuomotor task (without rotational perturbation) inside the scanner to familiarize themselves with the experimental environment (approximate duration of 10 min). Inter-trial intervals (ITIs) were randomly selected from 4 to 14 s in 2-s increments and generated from an exponential distribution (Burock et al., 1998). The sequences of ITIs were different for all participants. During each ITI, a white cursor was presented at the center of the screen, indicating both the fixation point and the initial cursor position. For each of the five runs, regardless of the condition, the trial started with the appearance of a target circle with a diameter of 1.3 cm on a dark background. The appearance of the target served as an immediate “go” signal; there was no preparation delay phase. Upon target appearance, participants were instructed to immediately reach for the target using the pen held in their right hand, with a maximum total response time of 1.5 s. The trial was considered a miss if they failed to reach the target within this time. To encourage faster responses, the cursor turned red if no movement was made within 800 ms. Visual feedback of the cursor trajectory was not provided during movement time. Upon completion of the trial, the final cursor position was displayed at a fixed distance of 9 cm from center of the screen for 500 ms, providing feedback strictly on the angular direction of the movement regardless of where the physical hand stopped.

### Experimental manipulations

According to the specific design of each run, different types of manipulations were applied to the target position and cursor movement (Figure 1). Within each trial, the target appeared in one of the following locations: (i) middle: 9 cm to the left of the center; (ii) high: 5.2 cm vertically above the middle position; or (iii) low: 5.2 cm vertically below the middle position. Meanwhile, one of the following three sensorimotor mappings was applied to the cursor movement: (1) a baseline mapping (0° rotational perturbation), in which the cursor movement was aligned to the hand movement; (2) − 30° rotational perturbation, in which the cursor movement was rotated counterclockwise (CCW) by 30°, requiring participants to make a hand movement tilted by +30° from the desired cursor direction (as illustrated in Figure 1A, “Run 1 & 2”, middle panel); or (3) + 30° rotational perturbation, in which the cursor movement was rotated clockwise (CW) by 30°, requiring participants to make a movement tilted by −30° from the desired cursor direction (as illustrated in Figure 1A, “Run 1 & 2”, bottom panel).

**Figure 1:**
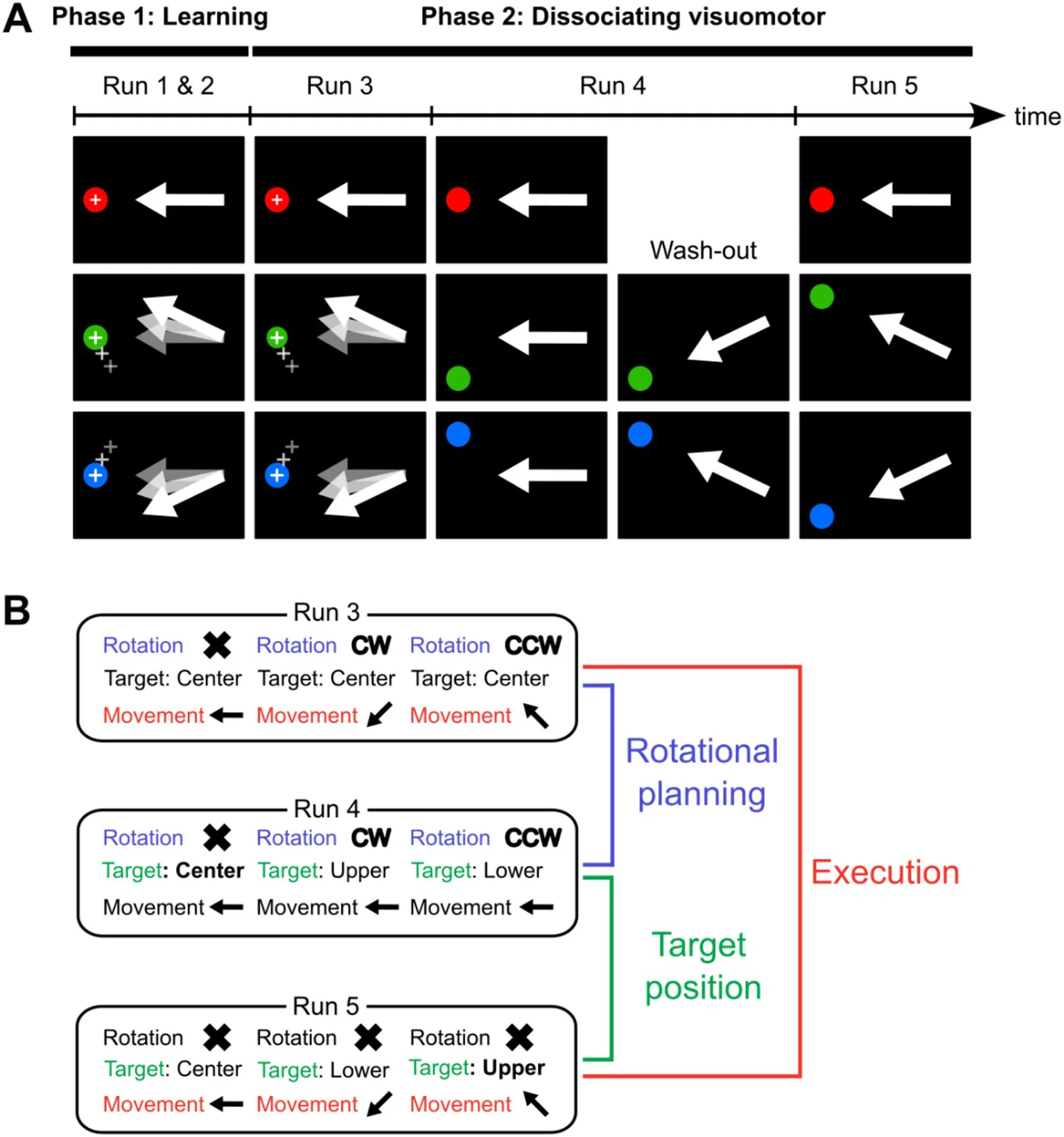
Experimental design isolating visuomotor components. Participants performed a visuomotor adaptation task across five functional magnetic resonance imaging (fMRI) runs. **(A)** During Phase 1, participants learned to counteract visuomotor rotations. Cursor rotations of 30° were applied, either clockwise (bottom panel) or counterclockwise (central panel). Participants used an MRI-compatible pen and a tablet to reach targets located on the left side of the screen, receiving feedback about the angular error at the end of each trial. The angular error was calculated as the deviation between the cursor’s final direction and the target’s direction. The first two runs were dedicated to learning the visuomotor rotations, while the last three runs were designed to separate visuomotor components. During Runs 1–3, targets were positioned horizontally at 9 cm to the left of the screen’s center, and visuomotor rotations were applied during adaptation trials. This design captures the neural activity related to both motor execution (reaching movements) and planning (rotational perturbations). Run 4 consisted of two phases: During the first phase, visuomotor rotations remained active to further assess rotational planning and visual target location. The second phase introduced a washout period of 24 trials, in which visuomotor rotations were removed to prevent interference from the learned visuomotor rotations in the subsequent run. In Run 5, participants performed reaching movements toward the targets while the visuomotor rotations were removed. We expect participants to accumulate information regarding the motor execution and visual target location. **(B)** Scheme of experimental design allowing separation of visuomotor components. Each run comprises two of the three visuomotor components, allowing for the use of a cross-validation scheme to dissociate the visuomotor components related to motor execution, rotational planning, and visual information processing about the target position.

In each run, distinct experimental conditions were defined by a unique combination of a target position and a specific sensorimotor mapping. Three different colored cues (red, green, and blue) were used to indicate these run-specific conditions. Target colors were counterbalanced across participants to account for any confounding effects. Furthermore, the presentation sequence of the experimental conditions involving CW and CCW rotations was also counterbalanced across runs and participants, with half of the participants starting with the CW rotation first and the other half starting with the CCW rotation first.

#### Phase 1: Learning to adapt to rotational perturbations (Runs 1 and 2)

In Runs 1 and 2, participants reached targets located in the same position (middle) but with different sensorimotor mappings applied (baseline, CCW, and CW). To identify each unique experimental condition, we adopted a [Target position]-[sensorimotor mapping] naming convention. For example, if the target position was in the middle and we applied a rotational perturbation of +30° (corresponding to the CW sensorimotor mapping), we would name that condition the Middle-CW condition.

Accordingly, the three distinct conditions in Runs 1 and 2 were defined as follows: (i) the Middle-baseline condition (target color: red; target location: middle; rotational perturbation: 0°; desired hand movement: horizontal); (ii) the Middle-CCW condition (target color: green; target location: middle; rotational perturbation: −30°; desired movement: tilted upward); (iii) the Middle-CW condition (target color: blue; target location: middle; rotational perturbation: +30°; desired movement: tilted downward).

Moreover, Runs 1 and 2 (each consisting of 144 trials) included three baseline blocks (12 trials each) that only contained the middle-baseline condition, and three pseudorandomly mixed blocks (36 trials each) that contained the Middle-baseline, Middle-CCW, and Middle-CW conditions. The baseline and mixed blocks alternated in a fixed order, with the baseline block first. The order of the conditions in the mixed blocks was pseudorandomized and identical for all participants. In each mixed block, the six possible sequences of the three conditions appeared once in each half of the blocks. Additionally, to avoid consecutive presentations of the same condition type, the last condition of each sequence differed from the first condition of the following sequence. These structures were identical for Runs 1 and 2, except that the presentation order within the mixed blocks was counterbalanced across participants.

#### Phase 2: Dissociating visuomotor components (Runs 3, 4, and 5)

While Runs 1 and 2 were designed to help participants learn different sensorimotor mappings, Runs 3–5 were deliberately designed to dissociate the visuomotor components, including motor execution, rotational planning, and visual information related to the target position.

##### Run 3

In Run 3 (108 trials), we kept the visual target position constant (Middle) while varying the applied sensorimotor mappings, creating conditions where the required physical movement (motor execution) directions and the decision-making processes (rotational planning) varied, but the visual input remained identical (Table 1 and Figure 1B, uppermost rectangle). Participants performed three conditions identical to Runs 1 and 2: (i) the Middle-baseline condition; (ii) the Middle-CCW condition; and (iii) the Middle-CW condition. The run consisted of single-condition blocks (12 trials each) presented in alternating schedules (e.g., a block of Middle-CCW trials, followed by a block of Middle-baseline trials).

**Table 1:** Experimental condition matrix.

Condition	Run(s)	Target color	Target position	Rotational perturbation	Required hand movement
Middle-baseline	1–5	Red	Middle	0°	Horizontal
Middle-CCW	1–3	Green	Middle	−30°	Upward
Middle-CW	1–3	Blue	Middle	+30°	Downward
Low-CW	4	Green	Low	+30°	Horizontal
High-CCW	4	Blue	High	−30°	Horizontal
High-baseline	5	Green	High	0°	Upward
Low-baseline	5	Blue	Low	0°	Downward

##### Run 4

Run 4 was divided into two distinct phases: a main experimental phase and a subsequent washout phase.

###### Main phase

In the first phase of Run 4 (108 trials), we manipulated both the target locations (Middle, High, Low) and sensorimotor mappings (baseline, CW and CCW) such that they offset each other. This required participants to plan distinct rotations for different visual targets while executing the exact same physical hand movement (horizontal) across all conditions (Table 1 and Figure 1B, middle rectangle). Participants performed three conditions: (i) the Middle-baseline condition; (ii) the Low-CW condition (target color: green; target location: low; rotational perturbation: +30°; desired movement: horizontal); and (iii) the High-CCW condition (target color: blue; target location: high; rotational perturbation: −30°; desired movement: horizontal).

###### Washout phase

Adaptation to visuomotor rotations typically results in strong aftereffects, which corresponds to strong deviations in movement trajectory opposite to the applied rotational perturbation when the perturbation is suddenly removed (Krakauer et al., 2006; Krakauer et al., 2000; Fernández-Ruiz and Díaz, 1999). We wanted to ensure that participants could perform the different conditions of Run 5 accurately from the very first trial, avoiding the need for a “de-adaptation” process. As such, the second phase of Run 4 comprised two washout blocks (24 trials) designed to eliminate any aftereffects of the rotational perturbations before starting Run 5. This washout phase was intended to revert the sensorimotor mappings to baseline, requiring participants to make direct movements toward the targets.

##### Run 5

In Run 5 (108 trials), all sensorimotor mappings were removed (baseline mapping only), meaning participants simply made direct physical movements toward varying visual target locations (Table 1; Figure 1B, lower rectangle). The conditions were defined as follows: (i) the Middle-baseline condition, (ii) the High-baseline condition (target color: green; target location: high; rotational perturbation: 0°; desired movement: tilted upward); and (iii) the Low-baseline condition (target color: blue; target location: low; rotational perturbation: 0°; desired movement: tilted downward). The run consisted of single-condition blocks (12 trials each) presented in alternating, pseudorandomized schedule.

### MRI acquisition

fMRI data were acquired using a 3 T Siemens Prisma scanner with a 64-channel head coil. The participants were provided with earplugs to minimize noise from the fMRI and foam pads to reduce head motion. Echo planar imaging (EPI) sequence was used to acquire functional scans (repetition time (TR): 2000 ms; echo time (TE): 35 ms; flip angle (FA): 90°; field of view (FOV): 200 × 200 mm; matrix: 100 × 100; axial slices: 72; and slice thickness: 2 mm; multiband acceleration factor: 3; in-plane GRAPPA acceleration factor: 2; partial Fourier: 1.0). A total of 410, 410, 309, 376, and 309 volumes were acquired in Runs 1 through 5, respectively. For anatomical reference, a whole-brain T1-weighted anatomical scan with an MP-RAGE sequence (TR: 2300 ms; TE: 2.28 ms; FA: 8°; FOV: 256 × 256 mm; matrix: 256 × 256; axial slices: 192; slice thickness: 1 mm; approximate acquisition duration: 6 min) was acquired before Run 3.

### fMRI data preprocessing

Imaging data were preprocessed using AFNI software (Analysis of Functional NeuroImages, NIH (Cox, 1996; Cox and Hyde, 1997); v17.3.00 / v24.0.17). Raw DICOM files were converted to 4D NIfTI format without removing initial TRs. Functional images underwent voxel-wise despiking (3dDespike) followed by slice-timing correction using 5th-order polynomial interpolation (3dTshift). Rigid-body head motion correction (3dvolreg) was referenced to the third volume of Run 3. Anatomical scans were skull-stripped and unifized (3dUnifize) before coregistration with functional images (align_epi_anat.py). The spatially registered images were then Normalized to the Montreal Neurological Institute 152 (MNI152) template (@auto_tlrc). To minimize spatial interpolation artifacts, transformation matrices for motion correction, coregistration, and standard space normalization were concatenated and applied in a single resampling step using cubic interpolation to 2 mm isotropic voxels (3dAllineate). All images were then spatially smoothed using a Gaussian kernel with a full width at half-maximum of 4 × 4 × 4 mm (3dmerge). Voxel timeseries were scaled from 0 to 200 with an average value of 100 to maintain consistency in signal intensity (3dcalc). Head motion metrics were calculated using Euclidean norm of movement derivatives (1d_tool.py) and TRs where framewise motion exceeded 0.4 mm, as well as the immediately preceding TRs were censored. Participants were excluded from further analysis if more than 15% of the volumes in any single functional run were censored.

### Univariate analysis for visuomotor components dissociation

Because our experiment was designed to separate the three visuomotor components of motor learning (motor execution, rotational planning, and visual target location), the preprocessed data for Runs 3–5 were analyzed separately using a general linear model (GLM; AFNI’s 3dDeconvolve), following the same procedure previously described for ROI definition. Each of these three imaging runs contained exactly three distinct experimental conditions. The three experimental conditions were defined as separate event-related regressors, where each trial was treated as a discrete 1.5-s event using a boxcar function convolved with a standard canonical HRF. All trials were included in estimating the regressors, regardless of whether the trial was successful (i.e., successfully reaching the target) or not. As there was no preparation delay at the start of the trial, the 1.5-s event regressor captured the entirety of the visual information processing, planning, and execution. The dissociation of these visuomotor components relied entirely on the experimental condition manipulations across Runs 3–5. Each of these runs included 3 experimental conditions, added as regressors to the GLM, alongside regressors of no interest modeling head movement (3 translations: x, y, z; 3 rotations: pitch, roll, yaw) and polynomial terms to account for fMRI signal drift. The first-level analysis resulted in one activation estimate (beta map) for each of the 3 conditions, in each imaging run.

To examine the neural substrates of the visuomotor components, two contrasts were defined at the individual participant level using the condition-specific activation maps (beta estimates) derived from Runs 3–5. We defined the directional contrast by comparing the CW and CCW sensorimotor mappings to assess directional differences in brain activity when planning and executing movement in response to opposing rotational perturbations. The adaptation contrast was defined by comparing the average of the two rotational perturbations against the unperturbed baseline (0° rotation) to examine the impact of introducing a perturbation. For Runs 3 and 4, these contrasts were specified based on the applied rotational perturbation. For Run 5, where all rotational perturbations were removed, the contrasts are based on the visual target location (upper and lower targets corresponding to the rotational perturbation conditions; middle target representing the baseline condition). For each of the three imaging runs independently, and for both contrasts, a group-level statistical *t*-map was generated across subjects using AFNI’s 3dttest++. To identify regions with significant differential activation between the contrasted conditions, the group maps were thresholded at uncorrected *p* = 0.001 and we determined the cluster-level family-wise error correction at α = 0.05. This resulted in thresholded binary maps of significant clusters for each run and contrast.

### Conjunction analysis

The general idea behind the conjunction analysis is to find common activation of brain regions between multiple conditions. By itself, a single within-run univariate contrast cannot independently dissociate the visuomotor components. For example, the Adaptation contrast (rotations versus baseline) tells us which brain region is implicated in processing visuomotor rotations, but it reflects an intermixed combination of motor planning, execution, and visual target processing. To dissociate those intermixed components, we employed a cross-run conjunction analysis that combined specific runs that shared only one of the three components (Figure 2A).

**Figure 2:**
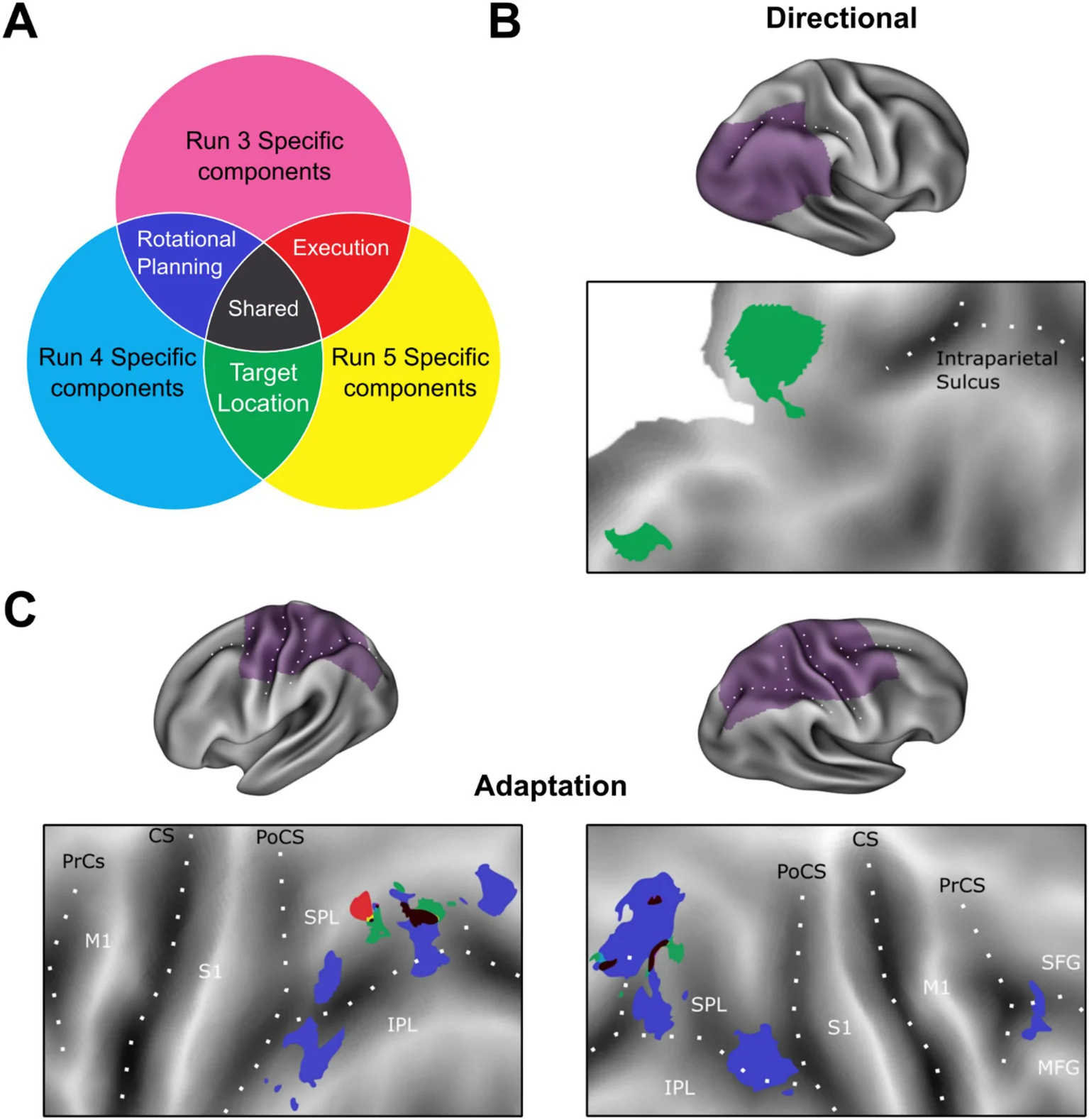
Univariate conjunction analysis reveals shared activation for visuomotor components in the posterior parietal and visual cortices. **(A)** Venn Diagram of the cross-validation experimental design. **(B)** Conjunction map displayed on a flattened surface of the right hemisphere in visual-related regions for the directional contrast with the inflated cortical surface above, highlighting the area of interest. **(C)** Cortical surface map of the left hemisphere (left panel) and right hemisphere (right panel) centered on the sensorimotor-related regions highlighted on the inflated cortical surface above for the adaptation contrast. Statistical maps are thresholded at uncorrected *p* < 0.001, cluster-level correction at α = 0.05. Shared activations are color-coded: red for execution, green for visual target location, blue for learning, and black for the three overlapping components. Major sulci are indicated in white dotted lines. CS, central sulcus; PrCS, precentral sulcus; PoCS, postcentral sulcus; PMC, premotor cortex; M1, primary motor area; S1, primary somatosensory area; SPL, superior parietal lobule; IPL, inferior parietal lobule; SFG, superior frontal gyrus; MFG, middle frontal gyrus.

We used the thresholded cluster maps from the univariate analysis to dissociate the neural substrates involved in motor execution, rotational planning, and visual information processing. This approach identified brain regions where multiple runs have overlapping activation. The intersection of significant clusters across the three imaging runs was calculated using AFNI’s 3dcalc function. The combined conjunction map was generated by assigning unique numerical weights to the binary cluster maps of Run 3, Run 4 and Run 5. By requiring a brain region to show significant task-related activation independently in multiple runs, this combined approach isolates the specific component shared between them (Figure 2A):

- Rotational planning: Identified by regions with overlapping activation in Runs 3 and 4. Because both runs required participants to counteract the exact same rotational perturbations but using different physical reaching directions with targets at varying positions, their conjunction isolates the neural substrates associated with planning to compensate the rotation.
- Motor execution: Identified by regions with overlapping activation in Runs 3 and 5. Because both runs required identical physical reaching directions (horizontal, upward, or downward) despite different rotational perturbations with targets at varying locations, their conjunction isolates the direction of the physical execution of the movement.
- Visual target location: Identified by regions showing overlapping activation in Runs 4 and 5. Because both runs presented the visual targets in the exact same spatial positions on the screen, their conjunction isolates visual information processing.

For both the directional and adaptation contrasts, we isolated the overlapping activations corresponding to each visuomotor component.

### ROI definition

To define visuomotor-related Regions of Interest (ROIs), we first determined regions with important, significant task-related activation throughout the brain using a GLM. To avoid circularity (double-dipping) for the conjunction and MVPA analyses, we computed these functional activation maps using only the first two runs (Phase 1) of each participant. The preprocessed time series for Runs 1 and 2 were provided together as input to AFNI’s 3dDeconvolve function but considered separate with intercept for each functional run. The three experimental conditions (Middle-baseline, Middle-CW, Middle-CCW) were defined as separate event-related regressors. Specifically, each trial was treated as a discrete 1.5-s event using a boxcar function convolved with a standard canonical hemodynamic response function (HRF). All trials were included when estimating the regressors, regardless of whether the trial was successful or not (reaching the target). These were added to the design matrix along with regressors of no interest modeling the head movement (3 translations: x, y, z; 3 rotations: pitch, roll, yaw), as well as polynomial terms to account for fMRI signal drift across the runs. This resulted in one activation map for each condition modeled. To identify brain regions with high overall task-related activation, we calculated the sum of parameter estimates across all three conditions. Using the combined activation maps for each participant, a group statistical t-map was generated across subjects [3dttest++ function (Cox et al., 2017)]. To identify highly activated regions, group statistical maps were thresholded at *p* < 0.001, uncorrected, and the family-wise error corrected *p*-value for the size of the activation cluster was determined using a permutation test (3dttest++ function with -Clustsim option).

To further determine our ROIs, we extracted predefined ROIs from the Automated Anatomical Labeling (AAL) toolbox (Tzourio-Mazoyer et al., 2002) and Brodmann atlas, provided in the MRIcro software (https://www.mricro.com). These predefined ROIs included the left precentral cortex (M1), left postcentral cortex (S1), bilateral supplementary motor area (SMA), right cerebellar lobule VI (CB6) and VIII (CB8), bilateral inferior parietal lobule (IPL), and bilateral superior parietal lobule (SPL). For the visual area (VIS), we used Brodmann areas 17 and 18. After resampling the anatomical predefined ROIs to match the resolution of the functional data, we defined our visuomotor ROIs by computing the intersection (logical AND) between the group statistical map and each of the resampled predefined ROIs, resulting in 10 distinct defined visuomotor ROIs.

### MVPA ROI analysis

To determine whether sensorimotor regions encode distinct visuomotor features, we employed cross-classification, a multivariate approach analogous to our conjunction analysis (Ogawa and Imamizu, 2013). Our design (Figure 1B) manipulated three components per run: execution (movement direction: up or 30°, middle or 0°, low or −30°), planning (visuomotor rotation: CW 30°, baseline 0°, CCW 30°), and visual target location (upper, middle, lower visual field). Each run contained a specific combination of these components, allowing us to train a classifier on one run and test it on another that shared the component of interest. For example, Runs 3 and 5 both required identical reaching movement directions, so cross-classifying 3 and 5 isolated execution-specific coding. Analogous run pairings also isolate planning or visual target location components (see Figure 1B for the pairings). Above-chance classification performance identified ROIs that contain selective, invariant representations for the component of interest.

We applied multivoxel pattern analysis (MVPA), a multivariate approach designed to detect differences in activation patterns across multiple voxels (Krasovsky et al., 2014), to compute decoding accuracies within the ROIs. To estimate the response amplitudes, we employed the Least Squares Separate (LSS) approach, which minimizes collinearity between adjacent trials (Mumford et al., 2012). For each individual trial, a separate GLM was constructed where the current trial was modeled as the primary regressor of interest (a 1.5-s event utilizing a boxcar function convolved with a standard canonical HRF), using spatially smoothed data. The model also included three separate regressors combining all other remaining trials in the run, split by their specific experimental condition. Six head-motion parameters and polynomial drift terms were included in every model. Missed trials were excluded from the analysis (see behavioral analysis for details). We converted beta estimates to *t*-values to down-weight the impact of high-variance voxels (Misaki et al., 2010). Our analysis focused on whether the directional and adaptation contrasts could be discriminated within the defined ROIs. However, for the Adaptation contrast, we restricted our analysis to the rotational planning component. Because MVPA relies on voxel-by-voxel pattern analysis, grouping distinct physical movement directions (motor execution) or distinct spatial target positions (visual target location) into a single, unified class is counterintuitive for decoding.

For classification, we used a linear support vector machine classifier (SVC) implemented in scikit-learn (Pedregosa et al., 2011) with default parameters (version 1.6.1, regularization parameter *C* = 1). The experimental design provided a natural two-fold cross-validation: train on run A and test on run B, and vice versa. Because for both contrasts, the number of trials in the training set was unbalanced, class weights were set inversely to class frequency to prevent bias toward the overrepresented class.

To assess statistical significance, we used a permutation test instead of relying on the theoretical chance level of 50%. For each ROI × component × contrast, we generated participant-specific null distributions by permuting class labels 2,000 times. We then bootstrapped 10^5^ group-level samples by resampling (with replacement) a single chance decoding accuracy per participant (*N* = 33) and averaged it to obtain a mean chance decoding accuracy. The mean decoding accuracy for each ROI was considered significant if it exceeded the 97.5th percentile of this distribution (two-tailed *α* = 0.05). To correct for multiple comparisons across ROIs, we controlled the family-wise error rate (FWER) using the Holm-Bonferroni correction (Holm, 1979).

### MVPA searchlight analysis

In addition to the ROI analysis, we used a searchlight analysis (Kriegeskorte et al., 2006) to compute decoding accuracy across the entire brain. We used *t*-values, as we did for the MVPA ROI, with a radius of 5 mm (93 voxels) and a linear support vector machine classifier (SVC) using the default parameters implemented in scikit-learn (version 1.6.1). After computing decoding accuracy for each visuomotor component at each searchlight within the brain volume, we used AFNI’s function 3dttest++ (Cox et al., 2017) to determine whether each voxel exhibits above-chance decoding accuracy. For statistical significance, we used uncorrected *p* < 0.001 with the Clustsim option to account for FPR in multiple comparisons at a cluster level, with a threshold of α = 0.05.

### Behavioral measure

We obtained the hand direction angle for each trial to ensure that the participants were learning the different visuomotor mappings. The measurements were determined as the angle between the first point and the endpoint of the trajectories drawn by the tablet for each trial. Missed trials that did not reach the target within the maximum movement time were excluded from the analysis. Because the rotated mappings of the different conditions (High-CCW and Low-CW) were presented in the opposite direction according to the counterbalanced group, the measurements of the second group participants were plotted as negative values when the average hand direction angle was visualized (Figure 3). For statistical analysis, we conducted one-sample t-tests comparing each participant’s mean movement angle against the respective target angle (30°, 0°, or −30°, depending on the condition).

**Figure 3:**
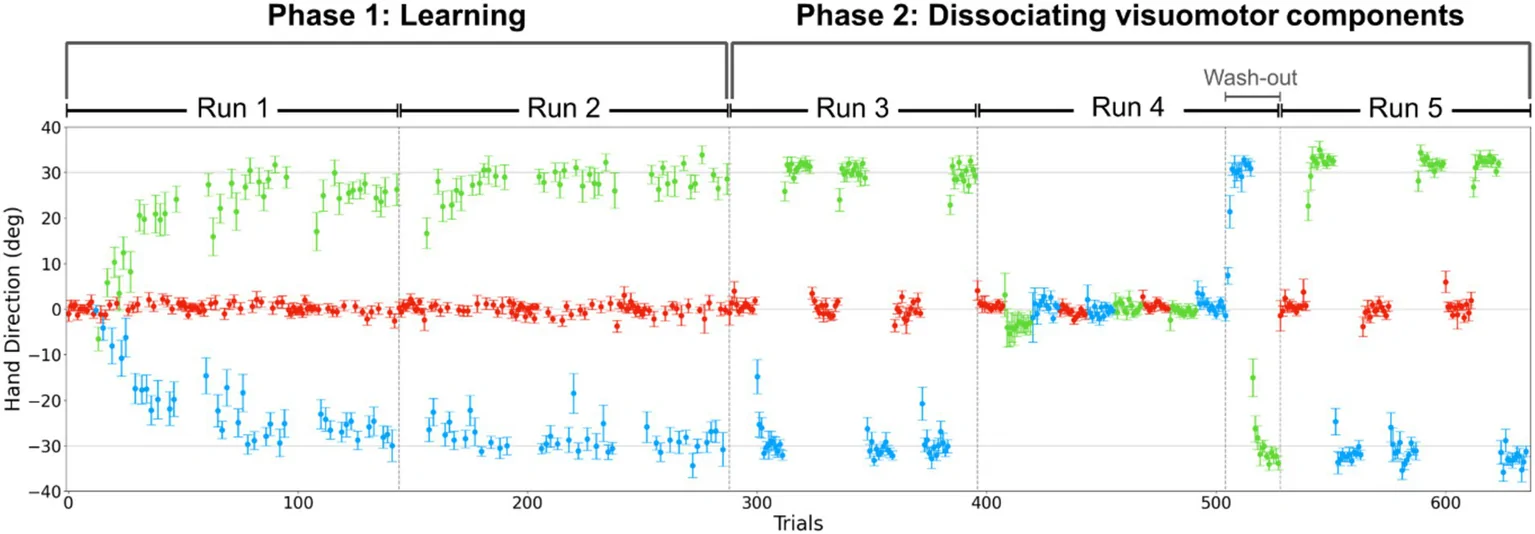
Participants successfully adapt to conflicting sensorimotor mappings and maintain goal directions across tasks. The average performance (mean ± SE) was measured as the hand direction angle (in degrees) across trials, with error bars representing the standard error across participants. Hand direction was calculated as the angle between the start and endpoint of the trajectory drawn by the participant on the tablet. Vertical dashed lines and top labels indicate the experimental phases (Phase 1: Learning; Phase 2: Dissociating visuomotor components), individual functional runs (Runs 1–5), and the washout period in Run 4. Horizontal dashed lines at +30°, −30°, and 0° indicate the ideal hand directions required for each experimental condition. The colors correspond directly to the experimental conditions shown in Figure 1: for Runs 1–4, they represent the contextual target colors associated with the different rotational perturbations, while for Run 5, they denote the target colors assigned to spatial locations.

## Results

### Successful adaptation of multiple visuomotor mappings

Thirty-three right-handed participants completed the visuomotor adaptation task. During each trial, participants used an MRI-compatible tablet and stylus to reach a circular target presented on the left side of the screen (Figure 1 and Table 1 summarize the experimental design and experimental condition manipulations). To distinguish the different combinations of target positions and rotational perturbations used throughout the study, we established a naming convention for the experimental conditions. Each condition is designated by its target location (High, Low, or Middle) followed by the applied rotation perturbation (baseline, CW or CCW). For example, a trial presenting the target in the central position (Middle) with a − 30° rotational perturbation (CCW) is referred to as the Middle-CCW condition. Hereafter, this naming method is used to describe the specific conditions presented across the different runs.

#### Learning to adapt to rotational perturbations

During the initial adaptation phase (Runs 1 and 2), three distinct visuomotor rotations of the cursor movement [0°, +30° clockwise (CW), and −30° counterclockwise (CCW)] were applied pseudorandomly. These rotations were paired with contextual color cues to denote three distinct experimental conditions: the Middle-baseline condition (red), Middle-CCW (green), and Middle-CW (blue) (Figure 1A). Participants fully adapted to the sensorimotor mappings throughout Runs 1 and 2, in which the different conditions highly interfered with each other due to their presentation in a pseudorandom order. Specifically, in the last block of Run 2, the movement directions were not significantly different from their “goal” directions (i.e., counteracting movements to the imposed rotations to reach the target). One-sample t-tests against the specific target angles confirmed that performance did not significantly deviate from the goals for the Middle-CCW (+30° reaching movement: mean ± SE = 29.06 ± 0.91°, *t*_(32)_ = −1.01, *p* = 0.321), Middle-CW (−30° reaching movement: mean ± SE = −29.05 ± 1.15°, *t*_(32)_ = 0.82, *p* = 0.421), or Middle-baseline (0° reaching movement: mean ± SE = 0.6 ± 0.69°, *t*_(32)_ = 0.86, *p* = 0.397) (Figure 3).

#### Dissociating visuomotor components

In the subsequent Run 3, the experimental conditions remained identical to those in Runs 1 and 2. However, to dissociate the specific visuomotor components rather than just continuing the sensorimotor mapping learning, discrete blocks of the conditions (12 trials per block) were used instead of a pseudorandom order. Participants successfully maintained the learned associations between the contextual cues for the conditions and the movement directions. Performance did not significantly deviate from the goal directions for Middle-CCW (+30° reaching movement: mean ± SE = 29.88 ± 0.64°, *t*_(32)_ = −0.19, *p* = 0.853), Middle-CW (−30° reaching movement: mean ± SE = −29.56 ± 0.56°, *t*_(32)_ = 0.77, *p* = 0.450), or Middle-baseline (0° reaching movement: mean ± SE = 0.70 ± 0.37°, *t*_(32)_ = 1.86, *p* = 0.072). Throughout Runs 1–3, participants successfully differentiated the conditions by gradually dividing the movements into three goal directions (Figure 3).

In Run 4, the participants were required to maintain hand movements in the horizontal direction (0°) across all three conditions while varying target locations (High, Low, and Middle). Participants successfully maintained movement angles near 0°, reaching the target regardless of the rotational perturbation applied, with no significant differences from 0° for Low-CW (mean ± SE = −0.91 ± 0.70°, *t*_(32)_ = −1.27, *p* = 0.214), High-CCW (mean ± SE = 0.57 ± 0.57°, *t*_(32)_ = 0.99, *p* = 0.328), or Middle-baseline (mean ± SE = 0.70 ± 0.38°, *t*_(32)_ = 1.81, *p* = 0.080). Finally, a brief washout phase was introduced at the end of Run 4 to avoid aftereffects. During this phase, the three conditions were differentiated solely by target position, meaning the ideal movement angle corresponded directly to the target location (Figure 1B). The actual movement gradually diverged into either 30° or −30° directions (High-baseline: mean ± SE = 31.11 ± 0.48°, Low-baseline: mean ± SE = −32.73 ± 0.37°).

In Run 5, following the washout phase, participants exhibited the expected patterns of movement angles for High-baseline (30° target: mean ± SE = 33.36 ± 0.55°, *t*_(32)_ = 5.99, *p* < 0.001), Low-baseline (−30° target: mean ± SE = −30.02 ± 0.52°, *t*_(32)_ = −0.04, *p* = 0.967), and Middle-baseline (0° target: mean ± SE = 0.94 ± 0.40°, *t*_(32)_ = 2.33, *p* = 0.026). Given participants’ successful performance, we assumed that Runs 3–5 reflected the acquired knowledge of the learned sensorimotor mappings.

### Conjunction analysis

To dissociate the neural substrates of motor execution, rotational planning and visual target location, we conducted a conjunction analysis using voxel-wise GLM on data from Runs 3–5. By identifying the spatial overlap in neural activation between specific pairs of runs, we can dissociate each shared component (Figure 2A). This approach isolated rotational planning (Runs 3 and 4 overlap), motor execution (Runs 3 and 5 overlap), and visual target location (Runs 4 and 5 overlap).

To investigate the BOLD response associated with these visuomotor components, we examined two whole-brain univariate contrasts designed to assess activation differences between the experimental conditions: (1) the adaptation-vs.-baseline contrast (CW and CCW rotations vs. 0° baseline) and (2) the directional contrast (CW vs. CCW). Note that for Run 5, which occurred after the physical perturbations were removed during a washout phase, the experimental conditions were defined entirely by the contextual color cues previously associated with those specific mappings (Figure 1A).

For the adaptation contrast, we identified significant BOLD activation corresponding to all three visuomotor components (Figure 2C). For the rotational planning component we observed significant overlapping clusters in the bilateral superior parietal lobe (SPL) and inferior parietal lobe (IPL), as well as in the right superior frontal (SFG) and middle frontal gyrus (MFG) (see Table 2 for detailed cluster extents). For both the motor execution component (conjunction of Runs 3 and 5) and the visual target location component (conjunction of Runs 4 and 5), significant overlapping clusters were found in the bilateral SPL. These results indicate that the BOLD responses were greater during the adaptation conditions compared to the baseline condition within the posterior parietal cortex (PPC) across all three visuomotor components, and within the frontal cortex, specifically for the rotational planning component.

**Table 2:** Conjunction analysis clusters.

Anatomical region	Peak (MNI)			Cluster size (voxels)
	*X*	*Y*	*Z*	
Directional				
Execution				
None				
Rotational planning				
None				
Target position				
R Cuneus	5	−101	7	611
R Lingual Gyrus	9	−81	−9	116
Adaptation				
Execution				
R Superior Parietal Lobule	21	−71	47	126
L Superior Parietal Lobule	−25	−71	51	120
L Inferior Parietal Lobule	−37	−55	51	20
Rotational planning				
L Inferior Parietal Lobule L Superior Parietal Lobule	−39	−43	35	852
R Superior Parietal Lobule	27	−73	37	557
R Inferior Parietal Lobule	35	−49	37	328
R Superior Frontal Gyrus	23	−1	45	93
R Inferior Frontal Gyrus	49	9	21	58
Target position				
R Superior Parietal Lobule	17	−71	45	205
L Superior Parietal Lobule	−25	−71	51	164
Shared				
R Superior Parietal Lobule	21	−71	47	126
L Superior Parietal Lobule	−25	−71	51	59

L, left; MNI, Montreal Neurological Institute; R, right.

For the directional contrast, the conjunction analysis identified two significant clusters specific for the visual target location component: the upper and lower regions of the calcarine sulcus in the right visual cortex (Table 2 and Figure 2B). In contrast, we found no significant overlapping regions for the motor execution and rotational planning components in the directional contrast.

### Multivariate pattern analysis (MVPA) with cross-classification

The core objective of our experimental design is to leverage multivariate pattern analysis (MVPA) to identify the specific neural substrates encoding the visuomotor components. After identifying brain regions with important BOLD activity (Figure 4), we first consider the directional contrast for the MVPA analysis (Figure 5). Although the whole-brain univariate conjunction analysis revealed no significant difference in BOLD signal amplitude between the two rotational perturbations, the MVPA revealed significant above-chance classification accuracy in the contralateral (i.e., left) M1/S1 (M1: 53.1%, *p*(unc) < 0.001, *p*(FWER) < 0.003, S1: 54.2%, *p*(unc) < 0.001, *p*(FWER) < 0.001). Furthermore, mean decoding accuracy was significantly above chance level in the left inferior parietal cortex (L IPL: 52.6%, *p*(unc) = 0.001, *p*(FWER) = 0.010), visual area (VIS: 53.0%, *p*(unc) < 0.001, *p*(FWER) < 0.003) and right cerebellar lobule VI (CB6: 52.6%, *p*(unc) < 0.002, *p*(FWER) = 0.010). These results indicate directional selectivity for the motor execution component in the sensorimotor regions, cerebellum, and visual cortex. Conversely, BOLD activity patterns for the execution of the reaching movement were not accurately decoded in the superior parietal cortex (L SPL: 51.7%, *p*(unc) < 0.028, *p*(FWER) = 0.133; R SPL: 51.4%, *p*(unc) = 0.053; R IPL: 50.8%, *p*(unc) = 0.180), the right cerebellar lobule VIII (CB8: 48.2%, *p*(unc) = 0.980) or the supplementary motor area (SMA: 49.7%, *p*(unc) = 0.612) (Figure 5).

**Figure 4:**
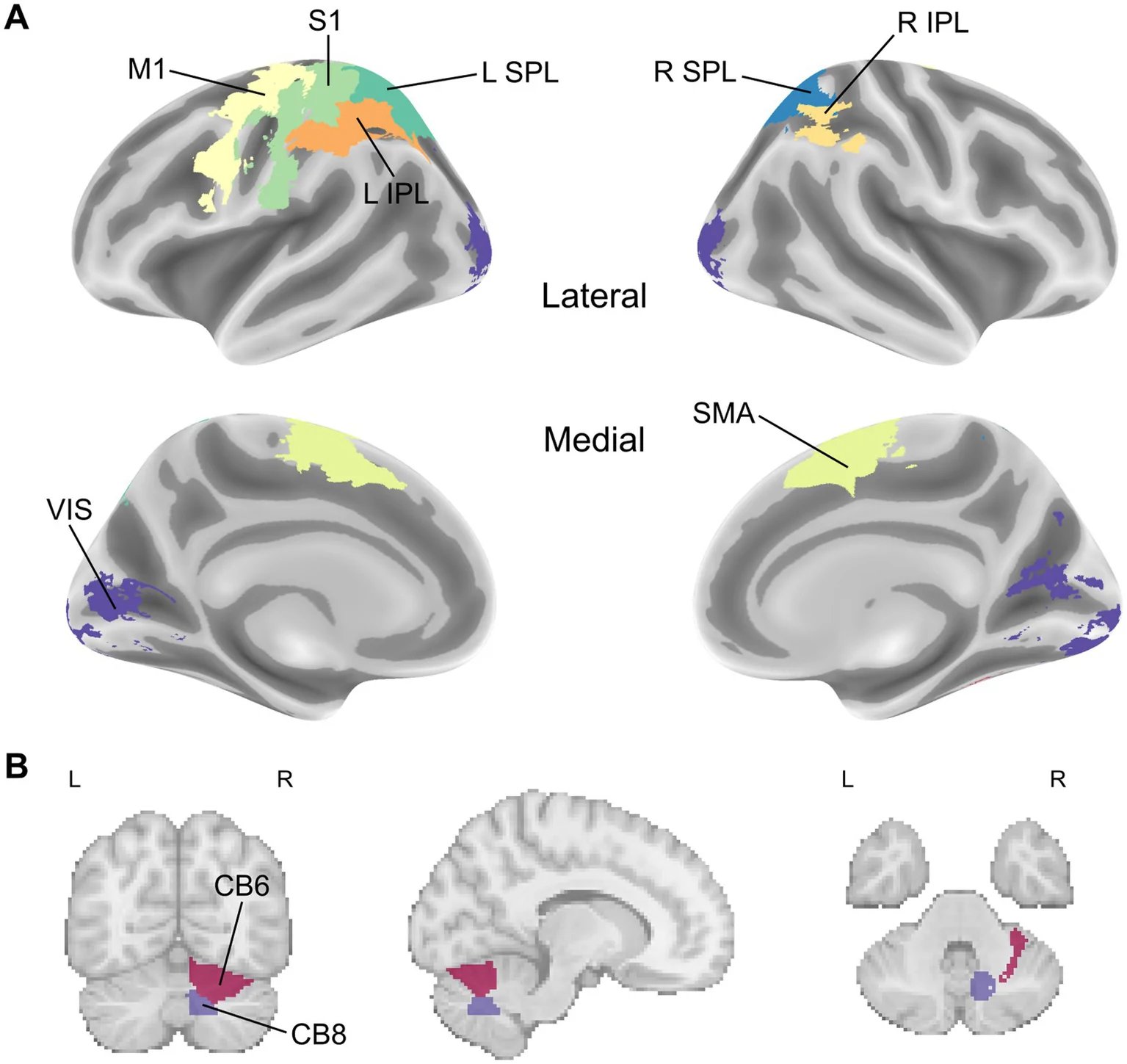
Representative brain areas of regions of interest. ROIs were defined as the conjunction of activation maps derived from the motor adaptation task (thresholded at uncorrected *p* < 0.001) and predefined ROIs from the AAL Atlas. **(A)** L, left; R, right; M1, left primary motor area; S1, left primary somatosensory cortex; SMA, supplementary motor area; SPL, superior parietal lobule; IPL, inferior parietal lobule; VIS, early visual area (Brodmann area 17 and 18). **(B)** CB6/CB8, right cerebellar lobule VI and lobule VIII, shown across orthogonal slices at MNI coordinates (x = 12, y = -65, z = -37).

**Figure 5:**
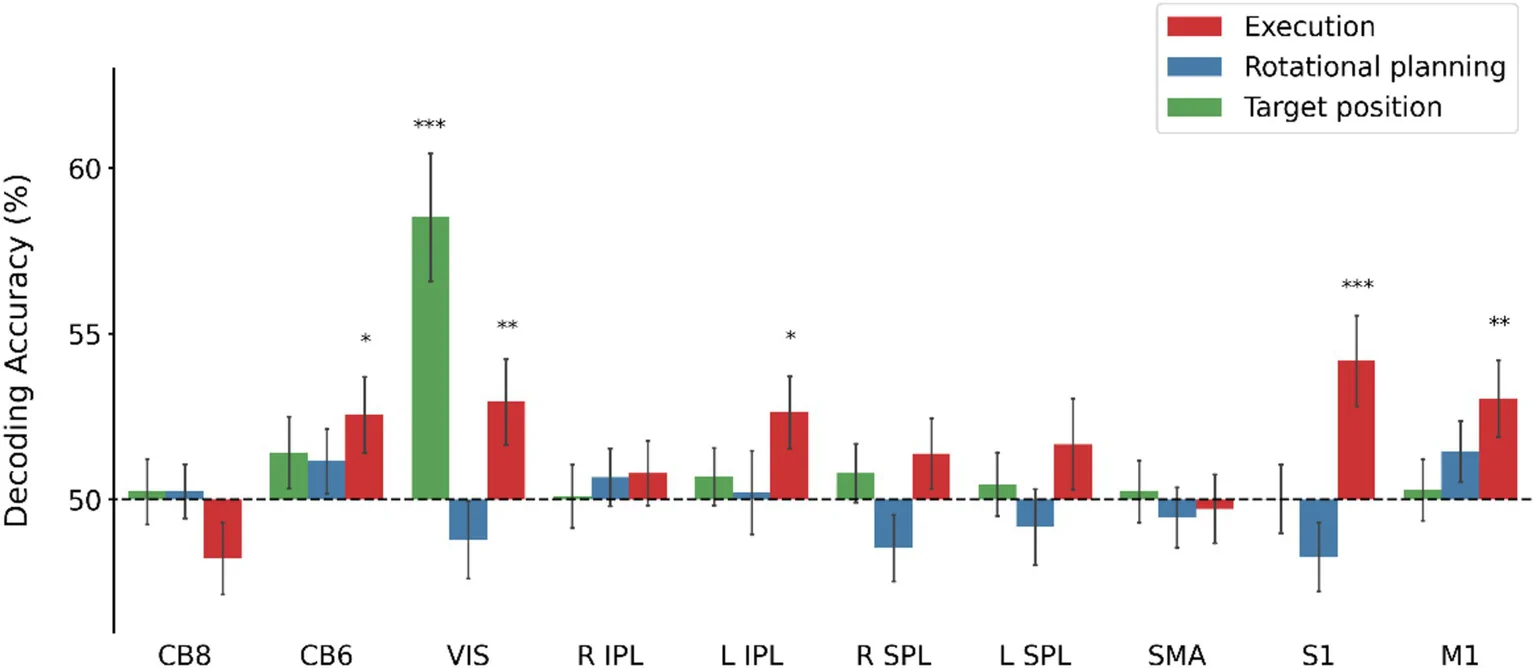
Directional reveals distinct neural representations for opposing motor execution and visual targets, but not rotational planning. We calculated the mean decoding accuracies for the directional contrast across participants using a cross-validation approach to dissociate visuomotor components for each ROI. Decoding was performed for the three visuomotor components: execution (red), planning (blue), and visual target location (green). The dotted line marks the theoretical decoding chance accuracy (50%, two visuomotor rotations), and error bars indicate SEM across participants. Significant above-chance decoding accuracy (randomization analysis, Holm-Bonferroni correction) is indicated with asterisks (* < 0.05, ** < 0.01, *** < 0.001). R (right), L (left), M1 (left primary motor area), S1 (left primary somatosensory area), SMA (supplementary motor area), SPL (superior parietal lobule), IPL (inferior parietal lobule), VIS (early visual area – Brodmann 17 and 18), CB6/CB8 (right cerebellar lobules VI and VIII, respectively).

For the visual target location component, the visual area exhibited the highest decoding accuracy (VIS: 58.5%, *p*(unc) < 0.001, *p*(FWER) < 0.001), confirming its strong selectivity for target position. No other regions exhibited significant above-chance decoding accuracy for this component (M1: 50.3%, *p*(unc) = 0.356; S1: 50%, *p*(unc) = 0.483; SMA: 50.2%, *p*(unc) = 0.392; L SPL: 50.5%, *p*(unc) = 0.307; R SPL: 50.8%, *p*(unc) = 0.168; L IPL: 50.7%, *p*(unc) = 0.212; R IPL: 50.1%, *p*(unc) = 0.435; CB6: 51.4%, *p*(unc) = 0.055; CB8: 50.2%, *p*(unc) = 0.393; Figure 5).

Unexpectedly, we did not find significant above-chance decoding accuracy for the rotational planning component in the cerebellum or any other predefined ROI. Specifically, the right cerebellar ROIs failed to discriminate between the neural plans made to counteract the CW and CCW perturbations (CB6: 51.2%, *p*(unc) = 0.086; CB8: 50.3%, *p*(unc) = 0.389). Moreover, no ROI showed significant above-chance decoding accuracy for rotational planning, including motor areas (M1: 51.4%, *p*(unc) = 0.051; SMA: 49.5%, *p*(unc) = 0.728), somatosensory and parietal areas (S1: 48.3%, *p*(unc) = 0.975; L SPL: 49.2%, *p*(unc) = 0.836; R SPL: 48.5%, *p*(unc) = 0.956; L IPL: 50.2%, *p*(unc) = 0.383; R IPL: 50.7%, *p*(unc) = 0.214), and the visual area (VIS: 48.8%, *p*(unc) = 0.921) (Figure 5).

### Classification of adaptation contrast for the rotational planning component

For the adaptation contrast (Supplementary Figure S1), we focused exclusively on the rotational planning component to evaluate the neural substrates of the sensorimotor mappings independent of their rotational direction. We found that the activity patterns within the sensorimotor ROIs successfully distinguished between conditions with and without rotational perturbations (Supplementary Figure S1). Significant above-chance decoding accuracy was observed across M1, S1, the bilateral PPC, the visual area, bilateral SMA and the right cerebellar lobule VI (M1: 54.1%, p(unc) < 0.001, p(FWER) < 0.001; S1: 54%, p(unc) < 0.001, p(FWER) < 0.001; L IPL: 53.3%, p(unc) < 0.001, p(FWER) < 0.001; R IPL: 52.3%, p(unc) = 0.001, p(FWER) < 0.002; L SPL: 52.5%, p(unc) < 0.001, p(FWER) < 0.002; R SPL: 52.7%, p(unc) < 0.001, p(FWER) < 0.001; CB6: 52.5%, p(unc) < 0.001, p(FWER) < 0.002; SMA: 52.4%, p(unc) < 0.001, p(FWER) < 0.002; VIS: 52.4%, p(unc) < 0.001, p(FWER) < 0.002). Meanwhile, the right cerebellar lobule VIII did not show significant above-chance decoding accuracy (CB8: 50.9%, p(unc) = 0.116). These results indicate that while the response patterns in these sensorimotor ROIs fail to discriminate between the specific direction of opposing perturbations (as shown in the directional contrast), they show selectivity for the presence of rotational perturbations during rotational planning in this experiment.

### MVPA searchlight

We conducted a whole-brain searchlight analysis (Kriegeskorte et al., 2006) using a searchlight sphere with a 5 mm radius, which included 93 voxels, to investigate neural selectivity for visuomotor components. We applied cross-classification to runs that shared common visuomotor components, assessed population-level decoding accuracy using voxel-wise one-sample t-tests, and corrected for multiple comparisons at the cluster level using permutation testing. Below, we report clusters that either overlap with our predefined ROIs or reveal additional task-relevant locations; the complete list of clusters for both contrasts is provided in Table 3.

**Table 3:** MVPA searchlight clusters.

Anatomical region	Peak (MNI)			Cluster level (*p*, voxels)	*z* value at peak	
	*X*	*Y*	*Z*			
Directional						
Execution						
L Primary Sensory Cortex	−45	−17	59	< 0.01/58	4.609	
R Middle Temporal Gyrus	55	−29	−9	< 0.01/57	4.550	
R Superior Parietal Lobule	19	−69	57	< 0.02/48	4.287	
R Lingual Gyrus	37	−83	−19	< 0.04/42	4.160	
L Superior Parietal Lobule	−21	−51	69	< 0.05/38	3.604	
Rotational planning						
None						
Target position						
Bilateral Occipital Cortex	3	−89	13	< 0.001/8,920	13	
Adaptation						
Rotational planning						
R Angular Gyrus R Inferior Parietal Lobule R Superior Parietal Lobule	45	−71	43	< 0.001/1,478	4.985	
L Inferior Frontal Gyrus	−53	17	21	< 0.001/350	5.071	
L Middle Occipital Cortex	−33	−75	25	< 0.001/225	4.502	
R Superior Frontal Gyrus	33	−3	61	< 0.001/192	4.866	
L Inferior Parietal Lobule	−59	−37	41	< 0.001/156	4.872	
R Supramarginal Gyrus	65	−21	29	< 0.01/84	3.523	
L Superior Frontal Gyrus	−31	−5	67	< 0.01/83	4.132	
R Superior Frontal Gyrus	25	69	13	< 0.01/80	3.569	
R Middle Frontal Gyrus	41	37	37	< 0.01/62	3.416	
R Inferior Frontal Gyrus	49	15	29	< 0.03/47	4.168	
R Middle Temporal Gyrus	55	−65	15	< 0.04/43	4.928	

L, left; MNI, Montreal Neurological Institute; R, right.

Because our ROI analysis was restricted to regions contralateral to the reaching hand, it did not probe the right hemisphere. The searchlight results for the directional contrast, therefore, complement the ROI-based findings (Supplementary Figure S2). Similar to the ROI-decoding analysis, the searchlight map in the left hemisphere highlights spatial selectivity in the S1 according to the direction of the reaching movement. At the same time, the visual cortex significantly decodes according to the location of the target. M1, however, showed no above-chance movement direction decoding. Additionally, the searchlight revealed a direction-selective cluster in the bilateral superior parietal lobule. For the adaptation contrast (rotational planning component only), searchlight decoding matched the ROI effects in the right SPL and bilateral IPL. It also detected additional clusters in the bilateral frontal gyrus and left occipital cortex (Supplementary Figure S3).

## Discussion

Humans possess a remarkable ability to adapt to dynamic environments, supported by neural systems capable of processing complex visuomotor tasks. Paradigms such as prism adaptation (Von Helmholtz, 1867), force field learning (Shadmehr and Mussa-Ivaldi, 1994), and visuomotor rotations (Krakauer et al., 2000) have been developed to investigate how humans adapt to novel or altered environments. Through these paradigms, it has been shown that the brain builds internal models to predict the sensory consequences of motor actions (Wolpert and Kawato, 1998). When a discrepancy arises between the predicted and actual movement outcomes, these internal models are updated through sensory prediction errors, with the cerebellum playing a critical role in this process (Taylor et al., 2010; Tzvi et al., 2020). In this study, we introduce a novel paradigm to investigate how humans learn conflicting motor tasks through visuomotor adaptation. Our approach employs a design inspired by statistical cross-validation to dissociate the neural representations of motor execution, rotational planning, and visual target location. By systematically varying target locations and rotational perturbations across runs, we leverage the overlapping experimental conditions to identify brain regions involved in rotational planning and execution and decode task-specific neural activity patterns using MVPA.

While behavioral performance confirmed that participants successfully adapted to the visuomotor rotations, throughout the experiment, we note a slight systematic bias toward the upper-field target (High-baseline) in Run 5. This bias may stem from the slightly awkward arm posture required to reach that position compared to the reaching movement needed for the target located in the lower part of the screen. However, this offset was negligible, an aiming angle of approximately 33.4° still placed the cursor on the target, leaving overall task performance unaffected.

Our univariate conjunction analysis revealed that the visual target location was the only component showing differential BOLD activity between opposite visuomotor rotations. We identified two clusters: one located above and one below the calcarine sulcus in the right hemisphere. Because the targets in the experimental conditions for Runs 4 and 5 appeared in opposite spatial locations (the upper-left and lower-left quadrants), this functional segregation of the visual field is consistent with prior studies (Schneider et al., 1993; Sereno et al., 1995; DeYoe et al., 1996). It aligns with the established role of the upper bank of the calcarine sulcus in processing information from the lower visual field and the lower bank of the calcarine sulcus in processing information from the upper visual field. Moreover, neural patterns in the visual ROI exhibited visual directional selectivity, depending on the target position on the screen, confirming distinct neural representations in the visual cortex for visual targets.

There was no difference in the overall BOLD activity in the sensorimotor areas between opposite rotational perturbations, as shown in the previous studies (Ogawa and Imamizu, 2013; Kim et al., 2015). Considering the current experimental design, the only important difference between the two adaptation tasks (CCW and CW) would be the opposite rotational perturbations applied. Thus, it is less likely that motor execution in different directions involves segregated sensorimotor regions, such as the somatotopic homunculus map (Penfield and Boldrey, 1937; Penfield and Rasmussen, 1950). Instead, sensorimotor regions may encode directional information at a finer level, which could be identified through the MVPA approach. Indeed, multiple studies show that neurons in the motor cortex are tuned to a preferred direction of movement, even for movements differing by up to 60° (e.g., between the +30° and −30° reaching angles) (Eisenberg et al., 2010; Fabbri et al., 2010; Truccolo et al., 2008) and remain tuned to their preferred direction even after visuomotor adaptation (Haar et al., 2015). Our MVPA analysis revealed BOLD activity patterns in sensorimotor regions, including M1, S1, the left IPL, and the ipsilateral cerebellar lobule VI, which exhibited directional selectivity for the direction of movement, even when the visual target location remained identical. Interestingly, we also found significant decoding accuracy for opposing directions of motor execution in the visual cortices (V1/V2, which correspond to Brodmann areas 17 and 18). Considering that visual imagery activates early visual areas, such as V1 and V2 (Kosslyn et al., 1993; Slotnick et al., 2005; Albers et al., 2013), participants may have been engaging in visual imagery, mentally simulating the movement of the cursor toward the visual targets. If this were the case, the activity in V1 and V2 could be attributed to imagined visual feedback from the movement rather than motor directional selectivity per se. Alternatively, it could reflect a visual feature confound. Voxel patterns in early visual areas (V1–V4) encode not only the visual boundaries but also the color of the stimuli (Brouwer and Heeger, 2009). Because color cues remained consistent with movement direction across both Run 3 and 5 (e.g., green for upward reach, blue for downward reach), the classifier in the visual areas may have separated the conditions based on low-level visual features.

While our experimental design included initial adaptation runs, the scope of our analysis focused on the learned sensorimotor mappings resulting from the adaptation process, rather than the adaptation process itself. From this, we found increased activity throughout the PPC in our univariate conjunction analysis for the rotational planning component when comparing rotational perturbation conditions to the baseline condition. This finding aligns with prior studies showing increased PPC activity during the late stages of visuomotor adaptation (Della-Maggiore et al., 2004; Krakauer et al., 2004), consistent with the timing of our experiment. Given the PPC’s established role in visuomotor adaptation, this activity is consistent with a role for the PPC in representing, retrieving, applying, or storing learned sensorimotor mappings (Della-Maggiore et al., 2004; Tanaka et al., 2009). In contrast, no significant activity was observed in the cerebellum, which is likely due to the participants’ advanced stage of learning. The cerebellum is known to play a critical role in predicting sensory errors (Blakemore et al., 2001; Tseng et al., 2007; Schlerf et al., 2012; Morehead et al., 2017; Tzvi et al., 2022), which is crucial during early stages of adaptation. However, since participants had already adapted to the rotational perturbations by this stage of the experiment, the number of errors was minimal. This absence of univariate activity may reflect the participants’ later stage of learning and reduced error levels. Tzvi et al. (2020) (Tzvi et al., 2020) found that activity in the right cerebellar lobule VI gradually decreased during adaptation to a visual rotation, which coincides with our results and the decrease in errors made by the participants (Figure 3).

Despite our expectation that distinct neural populations in sensorimotor regions would respond selectively to the rotational planning of opposing visuomotor rotations, we found no regions with significant decoding accuracy for this component in the directional contrast. This null result contrasts with previous studies (Ogawa and Imamizu, 2013; Kim et al., 2015), that successfully decoded rotational planning in sensorimotor regions, including M1/S1, SMA, the cerebellum and the posterior parietal cortex. The absence of decodable patterns in the PPC is particularly surprising considering its established role in storing updated sensorimotor mappings (Tanaka et al., 2009). Notably, those studies (Ogawa and Imamizu, 2013; Kim et al., 2015) demonstrated that movement kinematics (e.g., speed, mean, and error variance) could not account for the significant decoding results. However, unlike the current study, these earlier designs did not fully counterbalance all three visuomotor components (visual target location, motor execution, and rotational planning). Consequently, their decoding results may have partially reflected differences in physical hand movement direction, a confound our experimental design minimized. Alternatively, the relatively larger rotational perturbations in previous studies (Ogawa et al., 2013 (Ogawa and Imamizu, 2013): 90°; Kim et al., 2015 (Kim et al., 2015): 40°) compared to our study (30°) likely engaged more explicit cognitive strategies during rotational planning (Taylor et al., 2014), resulting in more distinct activation patterns in the sensorimotor ROIs. Lastly, the neural representation of rotational planning may be sensitive to the tool used to perform the task; earlier studies utilized a joystick involving wrist movements, whereas our design employed a tablet requiring whole-arm reaching movements. Future research is necessary to elucidate how varying degrees of rotational perturbations and different experimental tools affect the decoding of neural representations.

Our study presents three notable limitations. First, we included only three functional runs for the data analysis, resulting in a relatively small sample size (approximately 30 to 36 trials per condition) for robust multivariate decoding, especially within our cross-validation framework. While our experimental design provides a rigorous structure to dissociate the visuomotor components, this limited number of trials fundamentally constrains the statistical power of our analysis. Because rotational planning involves subtle neural activity, this sample size may lack sensitivity required to detect such spatial representations. Consequently, the non-significant decoding observed for the rotational planning component should be interpreted as a lack of robust decoding evidence, rather than definitive evidence for the absence of rotational planning representations within the predefined ROIs. Second, participants initiated their reaching movements as soon as the target appeared (without a preparatory interval), allowing the retrieval of the adaptation plan and the motor execution to overlap in time. As a result, the BOLD signal observed during the tasks reflects a mixture of retrieval and motor execution, which could prevent a clean dissociation of these two components. Our design limits the complete isolation of pure pre-movement planning. Future studies introducing an explicit preparation time using a Go / No-Go paradigm would separate planning from execution at the behavioral level (Ariani et al., 2022). It would also have been interesting to combine cross-classification with a Go/No-Go cue to obtain a within-run planning-only baseline. A third limitation concerns the potential contribution of external contextual cues to classifier performance. Although target color cues were counterbalanced across participants to prevent group-level confound, cue-condition mappings remained present across runs. While the visual area (VIS) decoding accuracy may partly reflect low-level visual features (color), it is unlikely to affect the decoding accuracy of M1, S1, CB6, or L IPL.

In summary, by dissociating the rotational planning and execution through our experimental design, we present new insights into the neural substrates underlying visuomotor adaptation. As expected, the primary visual cortex, the first cortical area to receive visual information, can distinguish spatial visual target locations, reflecting its well-established retinotopic organization. Furthermore, we found that after participants learned to counteract the rotational perturbations, the neural activity within the parietal cortex increased. This implies that this region is central to processing learned sensorimotor mappings following visuomotor adaptation. However, while the cerebellum and PPC (SPL and IPL) demonstrated decodable neural patterns for opposing motor execution, we did not observe significant decoding for opposing rotational planning. While this result seems contradictory to previous studies, and raises open questions about how distinct parameters of visuomotor mapping are represented in the human brain, we cannot rule out the possibility that our low trial count means this finding reflects a lack of robust decoding power rather than a definitive absence of rotational planning representations within the sensorimotor regions and PPC.

## Data Availability

The raw data supporting the conclusions of this article will be made available by the authors, without undue reservation. All MRI data used in this study are archived in the Hanyang University Network Attached Storage (NAS). Upon reasonable request, the corresponding author can provide all the dataset and code related to this study.
