# Supplementary material for: Dissociating neural representations of multiple learned sensorimotor mappings
Source: Front Behav Neurosci. 2026 Oct 15;20:1965610. doi: 10.3389/fnbeh.2026.1965610 (PMC13716789; doi:10.3389/fnbeh.2026.1965610)
Supplement: Supplementary file 1 [file Supplementary_file_1.docx]

Supplementary Material

**Supplementary Figure S1. Sensorimotor and parietal regions successfully encode the presence of rotational perturbations during planning.**

Mean decoding accuracies for the rotational planning (blue) component in the adaptation contrast across participants for each ROI. The dotted line marks the theoretical decoding chance accuracy (50%, two visuomotor rotations), and error bars indicate SEM across participants. Significant above-chance decoding accuracy (randomization analysis, Holm-Bonferroni correction) is indicated with asterisks (** < 0.01, *** < 0.001). Abbreviations: R (right), L (left), M1 (left primary motor area), S1 (left primary somatosensory area), SMA (supplementary motor area), SPL (superior parietal lobule), IPL (inferior parietal lobule), VIS (early visual area – Brodmann 17 & 18), CB6/CB8 (right cerebellar lobules VI and VIII, respectively).

**Supplementary Figure S2. Whole-brain searchlight decoding of rotational mapping, motor execution, and target position for the directional contrast.**

Whole-brain cross-classification MVPA searchlight analysis identifying cortical regions containing information for motor execution (red) and visual target position (green) in the directional contrast. Statistical maps are thresholded at a voxel-wise threshold of *p* < 0.001 uncorrected with a cluster-level threshold *p* < 0.05).

**Supplementary Figure S3. Whole-brain searchlight decoding of rotational mapping for the Adaptation contrast.**

Whole-brain cross-classification MVPA searchlight analysis identifying brain regions containing information for rotational planning (blue) in the Adaptation contrast. Statistical maps are thresholded at a voxel-wise threshold of *p* < 0.001 uncorrected with a cluster-level threshold *p* < 0.05).
